# “Food is Environmentally and Culturally Specific!”: A Preliminary Qualitative Study on U.S. Immigrant Parents’ Perceptions of School Lunch

**DOI:** 10.3390/ejihpe10010019

**Published:** 2019-11-21

**Authors:** Emmanuel Obeng-Gyasi, Godfred Antwi, Cecilia Obeng

**Affiliations:** 1 Department of Built Environment, North Carolina Agricultural and Technical State University, Greensboro, NC 27411, USA; eobenggyasi@ncat.edu; 2 Department of Public Health and Health Education, The College at Brockport, State University of New York, Brockport, NY 14420, USA; gantwi@brockport.edu; 3 Department of Applied Health Science, Indiana University School of Public Health, Bloomington, IN 47405, USA

**Keywords:** immigrants, food choices, immigrant foods, American foods

## Abstract

Children spend most of their day hours in school, so the dietary choices they make during school days are important for their childhood development and later life. This research examined food choices among immigrant families with school-age children in Indiana, USA. Open-ended questions were answered by 52 immigrant parents in 2017. Parents who answered the questions had children in classes ranging from kindergarten to grade 12. NVivo 11 was used for the initial analysis of the dataset, and several themes were identified. After the initial analysis, the data were categorized into major themes to condense the themes. Thirty-eight (73%) of the respondents indicated that their children ate school lunch, 14 (27%) indicated that they prepared lunch for their children to eat at school, and 39 (75%), mostly from non-industrialized countries, indicated that their children ate their home-country staple foods for dinner and on non-school days. Parents indicated that schools are serving the needs of immigrant children by serving varieties of foods during lunchtime.

## 1. Introduction

Food culture involves the methods for processing, cooking, and the consumption activity of food. Components of a food culture include activities such as the methods involved in obtaining, processing and preparing the raw materials, the cooking techniques in making the food, the setting of the dishes and place of eating, and eating habits [1]. Research findings consistently indicate that immigrants face challenges in their dietary habits on their arrival to the United States [2]. Studies show that acculturation and dietary habits have consistently been correlated with greater risk for obesity and chronic diseases [2,3,4]. In their study of family and social determinants of children’s eating patterns and diet quality, Patrick and Nicklas [5] noted that physical and social environment influence children’s eating patterns. Regarding the physical environment, they found that children are more likely to eat foods that are available and easily accessible to them. They also noted that children are inclined to eat greater quantities when they are provided with larger portions of food. Regarding the social environment, the authors noted that such socioeconomic and sociocultural factors as parents’ level of education, the constraints on the parents’ time as a result of work-related or other schedules, as well as their ethnicity or race, have some bearing on the types of foods that the children eat. Furthermore, the authors observe that mealtime structure, such as the social and physical characteristics of mealtimes, which included whether families eat together, watch television during meals, and the families’ source of foods (e.g., restaurants, schools), all impact children’s eating patterns. Finally, the authors note that parents’ behaviors, attitudes, as well as their feeding styles to a large extent affect their children’s eating patterns [5]. The authors conclude that interventions aimed at improving children’s nutrition must address the variety of environmental, physical, and social factors that impact children’s eating patterns [5].

On food choices by immigrant children in North America, studies such as those of Wang et al. (2016), Wang et al. (2011), Chen et al. (2011), Blanchet et al. (2017) [2,3,6,7], and many others have examined such important issues as food choices and food intake by Asian (Chinese, Korean, etc.), Latino, sub-Saharan African, and Caribbean immigrants living in the United States of America (USA) and Canada. The aforementioned authors have also examined food and/or dietary adaptation by the above-mentioned immigrants to the USA and Canadian food environments. Most of the studies mentioned above have focused on overweight and obesity, type 2 diabetes, and cardiovascular diseases found among immigrant (including refugee) children and adolescents. For example, in their study of 167 school-aged black immigrant children of sub-Saharan African and Caribbean descent’s lived experiences in Canada regarding dietary acculturation, Blanchet et al. (2017) [7], discovered that there were differences in the foods between Canada and those of their countries of origin. Specifically, using drawings by their studied participants, the authors discovered that whereas none of the top 10 foods drawn by the children from their countries of origin were highly processed, those drawn as being Canadian were highly processed. Top 10 foods identified in the children’s drawings as Canadian included pizza, juice and sugar-sweetened beverages, fries/poutine, and hamburgers. Apples were the most frequently mentioned food for Canada. However, foods identified by the children as being in the top 10 African and Caribbean foods were rice, chicken, meat, and leafy vegetables. According to the authors, emerging themes accounting for the food choices by the children were: Food availability and variety, mothers’ lack of time and fatigue, as well as the school eating environment. The researchers concluded that their studied participants experienced dietary acculturation. In particular, they noted that changes in dietary habits of the children mostly had a negative impact on their diet.

In another study by McCroy et al. (2017) [8] on the dietary patterns among Vietnamese and Hispanic immigrant elementary school children who participated in an after-school program, the authors, using the “My Food Choices” questionnaire designed by the Center for Pan Asian Community Services (CPACS) staff, examined the quality of food selection in a convenience sample based on their answers on a simple food intake questionnaire. The study questionnaire was made up of 54 questions, and each question asked about the frequency of consumption of a type or class of food, such as carrots, fried chicken, nuggets, or yogurt. The authors’ aim was to test for differences by gender and ethnicity. The quality of food selection was defined using consistency with the United States Department of Agriculture’s (USDA) Healthy Eating Pattern and dietary pattern analysis to determine which foods listed on the questionnaire were eaten in similar patterns. For example, they wanted to find out if there was any interconnectedness between say, chocolate candy consumption and cookies and cake consumption, or if rare consumption of apples correlated with avoidance of other fruit intake. The main finding was that there was generally an unhealthy diet in both ethnic immigrant children and that programs and interventions to improve diet quality were needed. Specific positive findings were that the studied children ate the recommended number of servings per week of fruit. In particular, half of the boys ate the recommended number of servings of vegetables and dairy foods, and the frequency of soda consumption was low with the median being only once per week and interquartile range being 0 to 3. However, 87% of the studied participants reported that their diets were below the weekly recommended number of servings for protein foods while 80% reported that their grain intake was below the weekly recommended number of servings. Furthermore, 60% reported that their dairy intake fell below the weekly recommended number of servings whereas 57% reported that their intake of vegetables fell below the weekly recommended number of servings. The authors found high levels of consumption of sweet snacks and fast food.

Of significance in the above study was the fact that the differences between the two ethnicities were statistically insignificant. However, the authors discovered that boys consumed significantly more servings per week of grains, protein, and fruits than girls. Most importantly, these study findings bolster those of Creighton et al. (2005), Lara et al. (2005) and Parks et al. (2005) [9,10,11], which also found relatively high fruit consumption and low consumption of vegetables and dairy by immigrants from Mexico and South Korea who had adopted American diets and eating patterns. Of concern is the fact that such adopted American diets, according to Cho et al. (2004), Jasti et al. (2011), and Yang (2007) [12,13,14], have the tendency to put such immigrants’ health at risk.

In McCroy et al.’s (2017) [8] study on the dietary patterns involving Vietnamese and Hispanic immigrant elementary school children who were participating in an after-school program, the authors noticed that the Vietnamese children had high consumption frequencies of fruits and vegetables and a low consumption frequency of dairy; these findings bolster earlier studies by Betts (1986), Weicha (2001), and Guerrero (2007) [15,16,17]. However, McCroy et al. (2017) [8] caution a comparison of their data with previous studies given methodological variations across the studies that included “differences in sample size, age groups, family income, acculturation status, and secular trends in U.S. food supply since the data across studies were collected from 1986 to 2013” (pg. 460). The researchers noted that because all the children in their study qualified for free school lunches, what they discovered as being the generally unhealthy diet of children in their sample could be attributable to low family incomes and not to the children’s ethnic backgrounds. Traditional Vietnamese and Hispanic diets, they noted, might be lower in protein and dairy foods than Western diets.

On gender, McCroy et al. (2017) [8] found differences in food consumption. Specifically, they discovered that boys reported more servings of proteins, grains, and fruits than did girls. They attributed this difference to behavioral and cultural factors. Specifically, they argued that there may be traditional cultural gender norms that favor boys and place higher value on boys’ and men’s good health over that of girls and women as reported in the U.S. Department of Agriculture, Agricultural Research Service (2016), and National Health and Nutrition Examination Survey (NHANES) 2013–2014 survey (see also Vlassoff, 2007) [18]. Another study on immigrant health and nutrition was that carried out by Visocky (2011) [19] in Tennessee, which examined the interconnectedness between dietary habits and health of recent Hispanic immigrants to Middle Tennessee. This study was done from an anthropological perspective, and the author’s aim was to find out: (a) how Latino and Hispanic immigrant populations’ nutritional statuses were affected by acculturation to the United States and to Middle Tennessee and (b) what factors motivated the changing of the immigrants’ own food practices. The study revealed that food insecurity and school lunches had a major impact on the immigrant children’s health. Another interesting discovery by Visocky was that the immigrants’ experiences were different for every group as well as for each individual. Thus, it was discovered that health outcomes of the immigrants differed based on their unique acculturation experiences. The author called for possible remedies to stamp out the declining health of the immigrants.

From the above studies, we observe that with the influx of new immigrants from Africa, Asia, Middle East, and Eastern Europe into Indiana, issues of acculturation, and more especially food choices and their potential impact on school age children’s health require scientific scrutiny. The aim of this study, therefore, was to examine food choices among immigrant families with school-aged children who live in Indiana. Specifically, the study sought to understand the immigrant parents’ reasons for determining whether the immigrant families would allow their children to eat school lunch and the role that the schools can play in satisfying the nutritional needs of the children.

## 2. Materials and Methods

### 2.1. Data Collection Method and Participants

Data were collected after approval from the last author’s institutional review board. Study participants were recruited with the help of an immigrant community leader who had extensive research experience and expertise in qualitative research. The process of recruiting participants included an initial invitation through word-of-mouth and distribution of flyers. Prospective participants who indicated their willingness to participate in the study were then briefed on the study’s objectives by the community leader after which, consent was obtained. A participant was deemed eligible to participate in the study if he/she had children in the school system and was a resident of the study area (Indiana). A total of 52 individuals met the inclusion criteria and thus were included in the final analysis of the study. The questions were composed of both closed and open-ended questions, and data collection lasted between August and December 2017. 

### 2.2. Instrumentation

The instrument used for this study was developed by two experienced qualitative researchers with a background in immigrant research and after reading articles such as Creighton et al. (2005), Lara et al. (2005), and Parks et al. (2005) [9,10,11]. The open-ended questions afforded the participants the chance to answer as they saw fit. Open-ended questions were used since they require careful selection of words by the researchers in order for the participants to grasp what is being asked [20].

A pilot study was conducted to test the reliability and validity of the study questions. Particularly, this was done to ensure that the study participants (immigrant parents) would understand the questionnaire. Ten participants were involved in the pilot study and were included in the main study because they answered the questions well. Thus, both the pilot and the main study used the same questions. Specifically, the questions included whether participants’ children ate school lunch and/or their home county foods. Additionally, participants were asked to provide their opinions on what schools could do to meet the nutritional needs of school-aged children. To include low-literacy parents or parents not fluent in English language in the study, the community leader helped explain the questions to speakers who had limited English competence. To ensure credibility and validity of the study, the researcher also asked participants to clarify and confirm what they had said to be sure that what they wrote was a true representation of their ideas.

### 2.3. Data Analysis

NVivo 11 allows a wide variety of data, including but not limited to documents, images, audio, video, to be organized and structured [21]. NVivo 11 was used for the initial analysis of the large dataset of the open-ended questions, and several themes were identified. The initial analysis using the NVivo 11 program generated several themes, and each of the authors independently categorized the themes into major combined themes with the view to condensing the already established themes. To confirm the combined themes and further strengthen the study’s credibility and internal validity, one peer scrutiny re-examined the identified themes. The peer scrutiny’s feedback provided a new perspective; in this case, it allowed the opportunity to step back and take a fresh observation at the combined themes and the peer comments into consideration (Shenton, 2003; Merriam, 2002) [22,23]. Re-checking themes in the data in this manner also helped to eliminate potential researcher bias, which ensured the accuracy of the results of the study. The content of the data was then organized according to common themes identified by the authors, and labels that fit participants’ narratives were given the same heading. Three themes emerged and are discussed under results.

## 3. Results and Discussion

There were 52 respondents; 11 males and 41 females. The majority of the respondents (46) were married. It was discovered that 51.9% (*N* = 27) of the respondents did not state their occupation. For those respondents who stated their occupations, their job types varied and included such work as housekeeping, lactation counselor, nursing assistant, warehouse manager, systems analysts, marketing personnel, business owners, community coordinators, and college professors. The majority of the respondents were from Mexico and such African countries as Nigeria, Ethiopia, Eritrea, Ghana, and Niger. Others were from China, India, Honduras, Colombia, Taiwan, Brazil, and Venezuela. See Table 1 below about respondents’ demographics.

### 3.1. Respondents’ Children Ate School Lunch

Those who indicated that they allowed their children to eat school lunch fell within seven thematic areas. The themes were: Lot of food varieties for the children; children would like to eat what their friends are eating; convenience; free of charge; school lunches are warmer; healthy; and children like it. The results are displayed in Table 2.

For some respondents, the reason for allowing their children to eat school lunches was because they believed that lunch served in the school consisted of a wide range or variety of food for the children to choose from. By providing a wide variety of foods, the children had the opportunity to avoid monotony in food choice; they also had the opportunity to try different nutritious foods if they chose “sensibly”. Food choices included grilled chicken Caesar salad, ham and cheese croissant, buffalo chicken salad, turkey bacon croissant, crispy chicken wrap, noodles, fruit yogurt cup, strawberry cup, beef nachos with cheese, and broccoli floret.

Some respondents indicated that their reason for making their children eat lunch was because their children would not eat the food they prepared for them since the children preferred to eat what their friends were eating at school. This, they indicated, lifted the burden of not being seen as part of the mainstream. It also helped to prevent a situation where the other children, who were unfamiliar with foods from their countries of origin, labelled their children and made fun of their foods.

For respondents who indicated that it was convenient for their children to eat lunch provided by the schools, they surmised that they did not have to shop and cook food for their children given that such children ate at school, and this gave them time to do other equally important chores at home for the benefit of their children.

Parents whose children ate lunch prepared by the schools because the lunch was free of charge noted that not spending extra money to pay for the lunch enabled them to save a little bit of money to take care of other bills and other necessities of life. The majority of the parents *N* = 29 in this category were in a very low-income job category, such as housekeeping, nursing assistant, or community coordinators, that worked up to three different low-paying jobs. The poor-income family category was defined according to the U.S. federal poverty threshold which, for example, considers $24,339 for a family of four with two children, $19,318 for a family of three with one child, or $16,543 for a family of two with one child to be in poverty [24]. The majority of the participants in this study fell within the poor-income family category as most were also eligible for free or reduced school lunch. One respondent expressed surprise that lunch was free for children from poor-income families in the United States and noted that she was comfortable with her child enjoying free lunch.

Some parents were very pleased about their children eating lunch prepared by the schools given that such lunches tended to be warmer compared to the food prepared at home and taken to school. As one participant noted, “having a warm meal helps eliminate the possibility of the child not eating the food”.

For the respondents whose reason for making their children eat school lunch was because they believed such lunches were healthy and the children loved such lunches and did not complain about them, we learned that they were satisfied with the conditions under which the school lunches were prepared and served. In fact, one parent noted that her daughter had nothing but good things to say about the school-served lunches. In a study examining parents’ perception of school lunch, the most frequent motivators were variety of foods, nutritional quality, and providing organic or sustainable foods [25].

### 3.2. Respondents’ Children Did Not Eat School Lunch

The reasons of respondents who indicated that their children did not eat school lunch included: Unsure about ingredients used in lunch preparation, children’s dislike of the school lunches, and excessive sugar and fat on the menu. Table 3 below presents results of participants’ decisions for not allowing their children to eat school-prepared lunch.

For parents whose children did not eat school lunch, the main common thread of argument that ran through their reasons was the fact that they claimed to want the “best for their children” and therefore wanted to be sure of the ingredients in the food that their children ate at school. Some respondents indicated that their children did not like the school’s lunch so to make sure that their children are eating during the lunch, they made sure to prepare food for the children to take to school.

Concerning the respondents who did not allow their children to eat school lunch because of the fear of there being too much sugar and fat in such foods, their common argument was that such foods as those described above were overly processed, not organic, and had the potential to contribute to their children becoming obese and potentially having other health problems in the future. Wildey et al. in a study of American middle schools found that schools sold primarily high-fat, high-sugar snacks and suggested interventions which included limiting sales of chocolate candy and substituting low-fat cakes, cookies, chips, and crackers instead [26]. Neumark-Sztainer et al. also spoke to this when they found that school food policies that decrease access to foods high in fats and sugars were associated with less frequent purchase of these items [27]. This indicates that immigrant parents hesitant to have their children eat school lunch may be more confident if the schools had policies in place to limit the purchase of such items.

On the question of whether respondents’ children ate their home country type of foods during non-school hours and days, a majority (*N* = 39) of the respondents indicated “yes.” Only three of the respondents answered that their children did not eat their home country style of food. Ten respondents (19%) indicated that their children sometimes ate their home country staple foods. 

Seventy-five percent of respondents, mostly from non-industrialized countries, indicated that their children liked home country staple food. They noted further that foods that originate from their home-countries are mostly organic and that they did not use sugar in preparing such foods. They added further that eliminating sugar from their foods made such foods healthy for their children, and they hoped that the schools would incorporate some of their suggested meals into their regular lunch programs to help their children eat foods that are similar to the food in their original country. A participant indicated that since “food is environmentally and culturally specific” adding food from their country will go a long way to help their children enjoy the food served to them by their schools.

With regards to the role that the schools could play in satisfying the nutritional needs of children, especially immigrant children, some respondents indicated that schools are already doing their best in meeting such needs and that their children are not complaining. This may have been due to parents being happy to have lunch available at school; thus, a decreased burden to have to make lunch, in addition to children being happy to be introduced to American food. Some respondents alluded to the environmental and cultural specificity of food and given that their children were in the United States physical environment and culture, one should compliment the schools meeting the nutritional needs of children. Other respondents offered suggestions as to how school lunch services could be improved. Particularly, some noted that schools could serve fresh vegetables and high-quality meats and salads at every meal time and avoid serving deep-fried foods. Such respondents called on the schools to cook healthy foods as much as possible. In a study by Kubik et al. they found that among both parents and teachers, 90% agreed that more healthy snacks and beverages should be available in schools [28].

This paper has highlighted the various ways in which schools are serving the needs of immigrant children. It also potentially indicates that schools’ lunch feeding programs exceed some parents’ expectations. Thus, a critical contribution of this study is that some Indiana schools may be serving the needs of immigrant children by serving varieties of foods during lunch time. Some respondents saw this important school program as being financially helpful to them because using their own resources to prepare lunch for their children daily would have been financially burdensome for them.

The studied immigrant families’ perception of the school lunch program was also discovered in this study. We learned that some saw the lunch program as being convenient for them given the fact that without it, preparing lunch each morning for their children before the children go to school would have been practically impossible, since some of the parents work a minimum of two jobs and must therefore leave home at the crack of dawn. They were most pleased that such a childhood nutrition program was available to their children in the United States; a service not available to their children in their original home countries.

In their study on children’s eating patterns, Patrick and Nicklas (2005) indicated that children’s physical and social environments influence their eating patterns; our research bolstered this view by demonstrating that the respondents believed that the school environment impacted their children’s food choices. Specifically, they noted that if they prepared food from home for their children, the children would not eat what they prepared for them because the children preferred to eat what their friends were eating at school [5]. The above-mentioned view regarding children’s preference to eat what others are eating reinforces Bandura’s Social Learning Theory (1977) [29], which indicates that environmental factors have the potential to shape behaviors.

Furthermore, as other researchers have pointed out, eating fatty food (Kobel et al. (2017), Wang et al. (2011), Wang et al. (2016)) [2,3,4] has steadily been correlated with greater risk for obesity and its associated health problems including chronic diseases. Our study indicates that parents’ fear of their children eating fatty and sugary foods contributed to their decision to prepare lunch for their children to avert the obesity problem. There is a critical need to assess disease in the school environment and strategies to combat them [30]. Lastly, parents’ suggestion of adding food varieties from different countries may be one way of diversifying the schools’ lunch and introducing children to different food cultures. Such a practice, though challenging to some extent, will help introduce the children to the global community and help make them tolerant and also prepare them for their future role in the global world.

### 3.3. Limitations

The study was conducted in the State of Indiana only, so the results and conclusions drawn may be relevant to the research participants’ environment only. The results therefore may not be generalizable to other immigrant populations across other states or regions. In addition, the food varieties identified by the immigrants that encouraged immigrants to allow their children to eat school lunch, may be solely liked by the immigrants who were a part of this study. Some parents in this study stated that school lunch was not available in their home countries; it is thus possible that any lunch provided at school exceeded their expectations. Nevertheless, the research findings documented in this study are significant and are of interest to a variety of researchers including those interested in the topic of immigrants’ health as well as researchers who want to work on school feeding.

## 4. Conclusions

This study has revealed the reasons behind immigrant parents allowing their children to eat or not to eat school lunches. The findings also indicate that some schools in Indiana are serving the needs of immigrant children by serving varieties of foods during lunchtime. This study’s findings could be used as a guide to include foods, especially organic-based ones and non-sugary foods from countries and cultures, that the students may otherwise not have the opportunity to experience in their schools. By so doing, the study findings have the potential to help give children access to a greater variety of foods to choose from during lunchtime.

### Implications for School Health

There is a high degree of cultural specificity concerning food types, ingredients used in food preparation, as well as the way and manner in which different foods are prepared and eaten. In view of the above-mentioned cultural specificities and the fact that most respondents were pleased with varieties of foods in school lunches in Indiana, it is suggested that in order to continue serving the needs of different children:

(A) Schools should invite parents from different cultures, especially parents from non-industrialized countries who are most likely to have foods unfamiliar to the mainstream USA children, to come to the schools to speak about how they prepare their unique meals, especially lunches. Where favorable conditions exist for a demonstration of how such foods are made, effort should be made to do such a demonstration.

(B) “Exotic” foods from different countries and cultures and the various ingredients used in preparing such foods could be brought to kindergarten classrooms during “show and tell” time for children to learn the names of the foods and ingredients to give them knowledge about different varieties of foods.

(C) When deemed necessary and appropriate, parents from different cultures could be invited to their children’s classrooms to make presentations about culture and food. The presentations could focus on the food ingredients used in different countries, food preparation, foods eaten during special occasions (e.g., festivals, holidays), what silverware (if any) are used in eating such foods, and the cultural significance of such foods in the lives of people who eat such foods. The above suggestion may help schools incorporate some of such dishes and subsequently improve upon the lunches that they serve in their dining halls at lunchtime.

(D) The schools could organize annual cross-cultural and intercultural food shows during which they could showcase efforts they are making at satisfying every child’s, but more especially immigrant children’s, nutritional needs. During these times, in addition to what the school is doing in this regard, students could also either bring already prepared meals to share with others or they could, with the help of teachers (and parents where necessary), show how dishes are prepared. Prizes may even be awarded to students, teachers, and parents who excel in these endeavors.

## Figures and Tables

**Table 1:** Descriptive profile of respondents by sex, number of years in the US, education, and number of children, (*N* = 52).

Characteristics	*N* (%)
**Sex**	
Males	11 (21)
Females	41 (79)
**No of years in the US**	
0–5	29 (56)
6–10	17 (32)
11–15	3 (6)
16–20	2 (4)
20+	1 (2)
**Education**	
Less than high school	6 (11)
High school graduate or G.E.D.	24 (47)
Technical school/associate degree	12 (23)
College degree (4 years or more)	6 (11)
Graduate/professional degree	4 (8)
**Number of children**	
1	3 (6)
2	20 (38)
3	21 (40)
4	3 (6)
5	4 (8)
6	1 (2)

**Table 2:** Parents reasons for allowing their children to eat school lunch.

Themes	*N* (%)
Lot of food varieties	10 (26.3)
Children would like to eat what their friends are eating	9 (23.8)
Convenience	5 (13.1)
Free	4 (10.5)
School Lunches are warmer	4 (10.5)
Healthy	3 (7.9)
Children like it	3 (7.9)
Total	38 (100)

**Table 3:** Parents reasons for not allowing their children to eat school lunch.

Themes	*N* (%)
Unsure of the ingredients in the food	5 (35.6)
Children did not like school lunch	5 (35.6)
Too much sugar and fat	4 (28.8)
Total	14 (100)
